# Insertions and the emergence of novel protein structure: a structure-based phylogenetic study of insertions

**DOI:** 10.1186/1471-2105-8-444

**Published:** 2007-11-15

**Authors:** Haiyan Jiang, Christian Blouin

**Affiliations:** 1 Faculty of Computer Science, Dalhousie University, Halifax, Nova Scotia, B3H 1W5, Canada; 2 Department of Biochemistry and Molecular Biology, Dalhousie University, Halifax, Nova Scotia, B3H 1X5, Canada

## Abstract

**Background:**

In protein evolution, the mechanism of the emergence of novel protein domain is still an open question. The incremental growth of protein variable regions, which was produced by stochastic insertions, has the potential to generate large and complex sub-structures. In this study, a deterministic methodology is proposed to reconstruct phylogenies from protein structures, and to infer insertion events in protein evolution. The analysis was performed on a broad range of SCOP domain families.

**Results:**

Phylogenies were reconstructed from protein 3D structural data. The phylogenetic trees were used to infer ancestral structures with a consensus method. From these ancestral reconstructions, 42.7% of the observed insertions are nested insertions, which locate in previous insert regions. The average size of inserts tends to increase with the insert rank or total number of insertions in the variable regions. We found that the structures of some nested inserts show complex or even domain-like fold patterns with helices, strands and loops. Furthermore, a basal level of structural innovation was found in inserts which displayed a significant structural similarity exclusively to themselves. The β-Lactamase/D-ala carboxypeptidase domain family is provided as an example to illustrate the inference of insertion events, and how the incremental growth of a variable region is capable to generate novel structural patterns.

**Conclusion:**

Using 3D data, we proposed a method to reconstruct phylogenies. We applied the method to reconstruct the sequences of insertion events leading to the emergence of potentially novel structural elements within existing protein domains. The results suggest that structural innovation is possible via the stochastic process of insertions and rapid evolution within variable regions where inserts tend to be nested. We also demonstrate that the structure-based phylogeny enables the study of new questions relating to the evolution of protein domain and biological function.

## Background

The majority of protein folds descend from a relatively small set of ancestral domains through divergent evolution [1-4]. The mechanism by which new structures emerge or evolve from existing proteins is still an open question. Unlike sequence evolution, the drift of the core of a domain structure is unlikely be stable and functional. Therefore, it is reasonable to postulate that structural innovation is more likely to be the result of evolution at the periphery of the conserved core of domains. A recent study about the indels in protein sequences found that the fraction of domains and individual unaligned regions increasing in size is almost twofold larger than the fraction decreasing in size [5]. Ancient domain families show bias towards insertions in the variable region which grow in size [5]. In comparison with deletions, a succession of insertions and rapid evolution appears to be a reasonable process that could lead to the emergence of novel protein architectures [2,6,7]. Determining the extent and the mechanism of emergence of protein structure is difficult because observations are limited to extant protein folds. The evolution of sub-structures over time must then be inferred from this limited information.

Because of a high degree of inter-residue dependence, protein structures evolve much more slowly than their sequences [8]. These structural constraints are relaxed in some parts of protein structures; these are typically surface loops where mutations, insertions, and deletions can occur with lesser consequences to the biological function for which a gene is selected. It was observed that the probability of observing insertions and deletions in a pairwise alignment of protein sequences correlates with their evolutionary distance [9,10]. The study of structural similarity of loop regions in homologous proteins also observed a linear correlation between sequence and structural similarities [11]. These observations are consistent with the assumption that insertion and deletion events are continuous processes that parallel the better characterized process of substitution.

Incremental change in loops through insertion or deletion is a possible mechanism that can generate new polypeptidic folds [6]. The proposed model of emergence assumes that regions with faster evolutionary rates, such as surface loops, are the most probable locations for the occurrence of rare events. As proteins evolve, insertions can accumulate in these loop regions without affecting the folding of the core structure. Unless an insert is eliminated by purifying selection, multiple and nested insertions will make surface loops appear to grow over time. These variable regions have the ability to explore the conformational space and thus acquire novel sub-structures. Subsequently, a fraction of novel structural features generated via this process can be positively selected, and eventually become independently folding units (e.g. domains).

The key element leading to structural emergence under the proposed model is thus the extension of surface loops into nascent substructures. Testing this model requires to infer the sequence of events (phylogenies) leading to structural variability in protein structures. A series of efficient and robust tools were developed to produce structure-based phylogenies of protein domains and high quality structure-based multiple sequence alignments. Phylogenetics usually relies on the signal in biological sequences to infer the evolution of a gene. The tertiary structure of a protein evolves much slower than its sequence, and potentially contains a phylogenetic signal which is likely to persist beyond the timeframe where sequence signal becomes saturated.

The structure-based phylogenetic method utilizes a distance measure *Q*_*H *_[12,13] to compute trees using the Neighbor-Joining algorithm [14]. We also developed a method to build the structure-based multiple sequence alignments. This tool is derived from a multiple sequence alignment method proposed by Casbon and Saqi [15], which generates multiple structure-based alignment by running T-Coffee [16] to perform hierarchical alignment using information from the pairwise structural alignments. In this work, we used the application Flexible structure AlignmentT by Chaining Aligned fragment pairs with Twists (FATCAT) [17] to produce the pairwise alignments. According to the results presented in this work, the trees inferred with *Q*_*H *_distances are consistent with results of sequence-based methods. The structure-based phylogenetic method is efficient and robust. Because the sequence identity amongst domain families is often low, the structure-based phylogenies are also more suitable for this study than sequence-based phylogenetic methods.

This work used the structure-based phylogenies to infer a possible sequence of insertion events leading to the extant domain structures. The objective was to assess whether complex and novel protein structures can arise through the incremental growth of variable regions in protein domains. The analyses were performed on a large test set of homologous proteins built from Structural Classification of Proteins (SCOP) database [18]. The study revealed a large portion of insertions are bounded by earlier insertions in the variable regions of protein domains. We demonstrate that the average size of inserts created by nested insertions is substantially larger than block inserts. We analyzed the conformations of inserts, and found some structures of nested inserts show complex or even domain-like fold patterns including helices, strands and loops. The β-Lactamase/D-ala carboxypeptidase domain family was used as an example to illustrate the inference of insertion events, and how the incremental growth of a variable region is capable to generate novel structures.

## Results

### Statistics of insertions

The results of the quantitative study of insertions are listed in Table 1. In the test set, 98% of domain families belong to the four SCOP classes all α, all β, α/β, and α+β. The detected insertions are not evenly distributed in the four classes. The amount of insertions in all β class is significantly larger than all α class. All α class has 97 families, 753 domains, and 796 inserts. For all β class, there are 114 families, 937 domains, and 1788 inserts. The size of all β class is slightly bigger than all α class. Whereas the inserts detected in all β class are almost twice as many as the inserts of all α class. The numbers of insert-containing regions per domain for all α, all β, α/β, and α+β classes are 1.1, 1.9, 3.1, and 1.5, respectively. The results indicate that domains from SCOP class α/β have more variable regions on average than those from other three classes.

**Table 1:** Statistics of insertions and nested insertions in the test set

	SCOP Classes						
		
	All α	All β	α/β	α +β	Multi-Domain	Membrane^g^	Total
*N*_SF_^a^	48	54	57	55	4	4	222
*N*_FA_^b^	97	114	131	96	5	4	447
*N*_domain_^c^	753	937	1159	677	27	17	3570
*N*_FA_NI_^d^	30	59	83	35	3	1	211
*N*_I_^e^	796	1788	3576	1034	121	41	7356
*N*_NI_^f^	158	339	1090	192	17	5	1801

^a ^Number of superfamilies; ^b ^Number of families with at least 3 domains; ^c ^Number of domains in the families with size ≥3; ^d ^Number of families with nested inserts; ^e ^Number of insert-containing regions; ^f ^Number of nested inserts; ^g ^Membrane and cell surface proteins.

Our method detected 7356 insert-containing regions, including 5555 block insertions and 1801 nested insertions. There are a total of 9691 insertions with size >1 observed in the 7356 inserts, in which 5555 are block insertions and 4136 insertions are nested. A total of 211 out of 447 families contained lineages with nested insertions. Consequently, our analysis of SCOP family test sets found that 24.5% of all insert-containing regions have at least one nested insertion while the remaining proportion are inserts that appear to have been inserted in a single step (block insertion). Overall, 42.7% of the inferred insertion events are those nested into an insert region. The addition of more homologous domains in a given family is likely to divide some of the block insertions into nested insertions. This, in turn, will increase the number of observations of nested insertions, although it is impossible to determine how much this would affect the net proportion of these two types of inserts.

**Figure 1:**
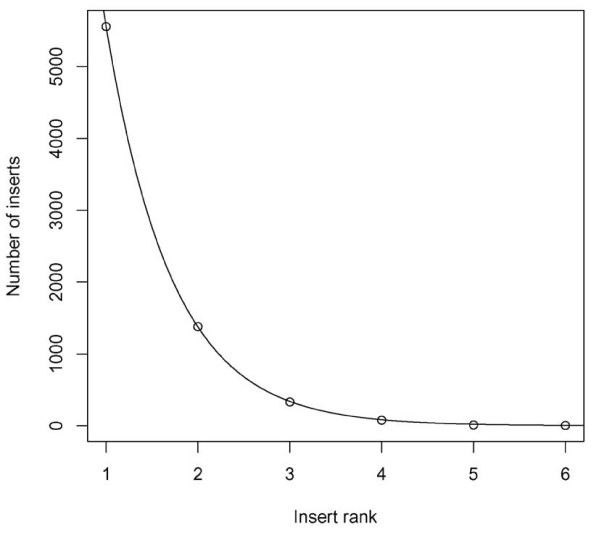
**Distribution of insert rank**. The function of the fitted exponential curve is *y *= *ae*^-*bx*^, *x *> 0, in which *a *= 22493.7, and *b *= 1.4.

**Figure 2:**
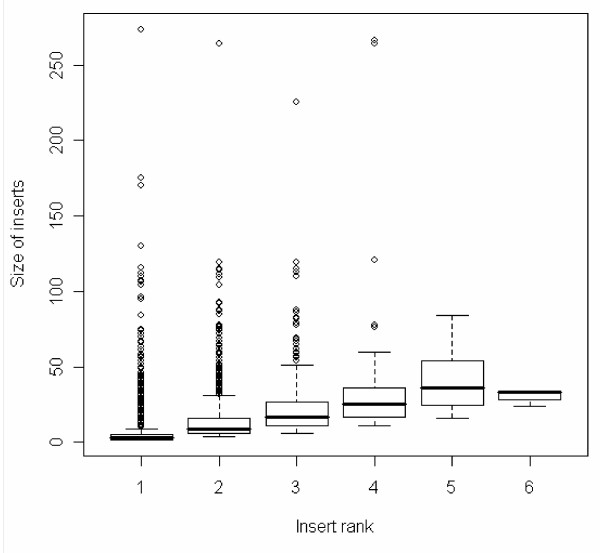
Box plot of the size for inserts with insert rank ranging from 1 to 6.

The distributions of insert rank and size were analyzed. The distribution of insert rank of the insert-containing regions fits an exponential decay (Figure 1). The total numbers of inserts with rank from 1 to 6 are 5555, 1378, 331, 77, 11, and 4, respectively. Four domains have maximal insert rank 6. They are domains d1gw0a3, d1aoza3, and d1gska3 from the multidomain cupredoxins family, and d1qhoa4 from the amylase family. Figure 2 shows the distributions of insert size with insert rank. The average sizes of inserts with rank ranging from 1 to 6 are 5.5, 14.3, 23.8, 35.9, 42, and 31, respectively. Despite the average size for inserts with rank 6, which were calculated from a small portion of samples, the insert size tends to increase with the insert rank. The observations are consistent with the model that variable regions of a protein appear to "grow" because of the accumulation of nested insertions. The inserted protein segments generated by the nested insertions are much longer than the block inserts. The average size of nested inserts with rank >1 is 17.2 residues long, whereas the average size of block inserts is 5.5. It indicates that block insertions, by virtue of their length, are less likely to be the site of structural innovation.

### Insertions produce complex sub-structure

To investigate whether insertions can create novel protein structure, structural complexity and novelty analyses were performed on a large dataset of protein domains. Our functional definition of complexity is when the p-value of the alignment of a segment to itself is below a significance threshold. Our functional definition of novelty is when a segment is complex and does not align with any other polypeptide segments other than itself. The screening of all identified inserts identified 77 complex block inserts and 80 complex nested inserts. The properties of the complex inserts are given in Table 2. The complex inserts were classified by their secondary structural content as Helices, Helices+Strands, Strands, and Loops. In comparison to the nested inserts, the secondary structures of the complex block inserts have more Loops and less Helices+Strands local conformations. The average size of the complex block inserts is 41 residues, which is smaller than the average 53 residues of the complex nested inserts. By comparing the complex inserts with domains from other superfamilies in the ASTRAL SCOP 1.69 subset, 17 out of 77 complex block inserts were identified as novel structures, and 30 nested inserts were identified as novel out of a set of 80 complex nested inserts. The nested insertions produced about twice as many novel structures as the block insertions. This is in part because nested inserts are averagely longer, and because stabilizing substitutions can accumulate between insertion events to stabilize the intermediate structures. It should be noted that some observed large complex 'block' inserts could be the product of nested insertions but there were not enough structures in the analysis to permit their identification.

**Table 2:** Properties of the complex block inserts and the complex nested Inserts

	Secondary Structure						
				
Complex Inserts	H^a^	S^b^	HS^c^	L^d^	Total	Average Length	Novel Structure
Block	30	5	19	23	77	41	17
Nest	34	7	26	13	80	53	30

^a ^H: helices; ^b ^S: strands; ^c ^HS: helices and strands; ^d ^L: loops.

**Figure 3:**
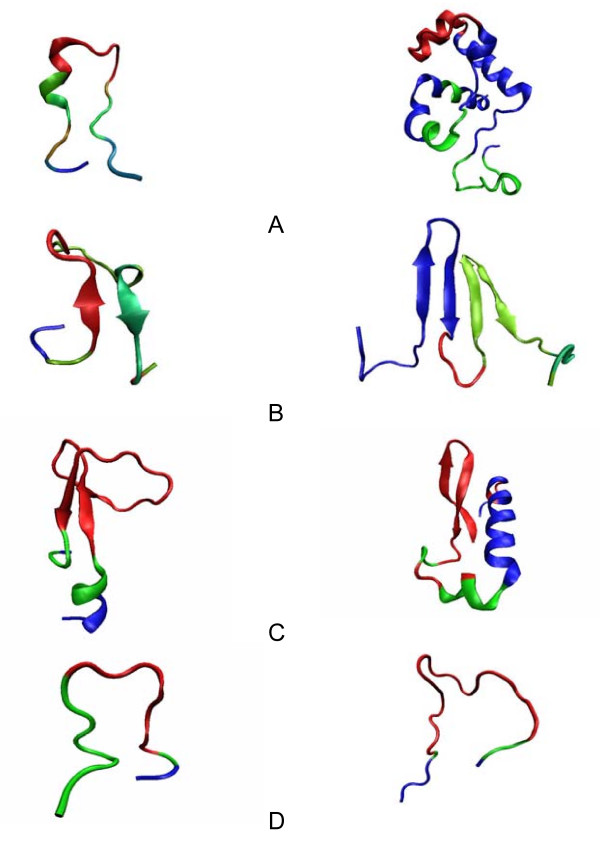
**Representative complex nested inserts**. A. Helices: d1aoza3(454–477) and d1j18a2(176–263). B. Strands: d1p99a_(157–183) and d1h6da1(320–374). C. Helices+Strands: d1r85a_(322–355) and d4lipd_(213–262). D. Loops: d1qo8a1(43–59) and d1v88a_(74–98). The insert ranks are color-coded from the most recent inserts (red), through shades of green, to the first insert event relative to the domain family root (blue).

Several representative inserts (insert region only) are shown in Figure 3 as examples of the structures produced by incremental growth through nested insertion events. The insert structures were colored with the mapping method described in the methodology section. The color, scaled from blue to red, indicates the insert rank to which each site is classified. For the colored inserts, the more blue intensive color implies the earlier the ancestral insertion event. This illustrates how incremental growth within variable region is capable to generate complex structural patterns.

It is much more difficult to demonstrate that existing domains have emerged via this mechanism. Evidence that a given domain in a gene is significantly similar to an insert-nesting variable region in an unrelated gene would constitute a demonstration that these novel folds can be propagated through the proteome. However, it is fair to assume that most common domains are relatively ancient and that structural intermediates are thus unlikely to exist to this day. The analysis of distant but evolutionarily related protein folds may provide further insight into this possibility.

### An example of the growth of variable region via stepwise nested insertions

**Figure 4:**
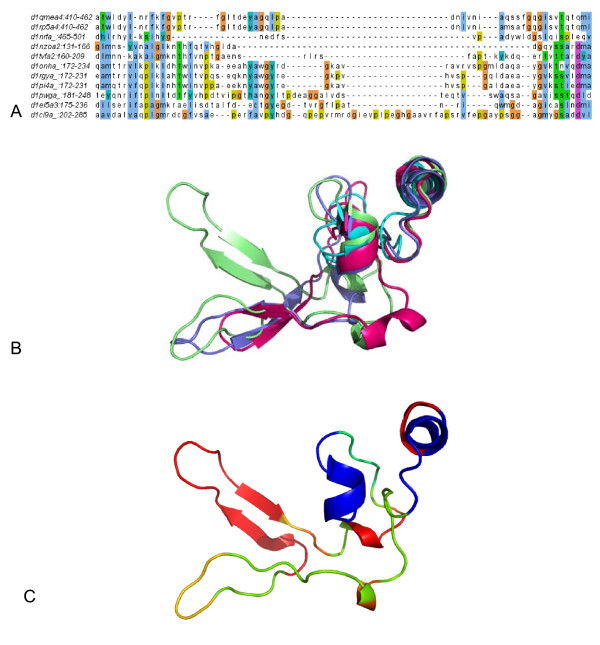
**Stepwise nested insertions detected in the esterase domain d1ci9a_ within the beta-Lactamase/D-ala carboxypeptidase family**. A. Multiple sequence alignment of the variable region. B. Multiple structural alignment of the representative variable regions from the domains: d1nrfa_ (cyan), d1onha_ (pink), d1ei5a3 (lavender), and d1ci9a_ (light green). C. The variable region of domain d1ci9a. Inserts are color-coded according the insert rank.

Figure 4 gives an example of the incremental insertions observed in the β-Lactamase/D-ala carboxypeptidase family (The sequence based phylogenetic tree is provided in additional file 1.  Additional Files 2 and 3 give another example of the nested insertions detected in the domain d1m56b1 within the Periplasmic domain of cytochrome c oxidase subunit II family). This family has been studied before with structure-based phylogenetic method because sequence similarity amongst these domains is too low to be aligned with confidence [19]. The domain d1ci9a_ is a novel esterase isolated from Burkholderia gladioli (EstB) with high deacetylation activity on cephalosporins [20]. Unlike other esterases, the protein is homologous to penicillin binding proteins, notably class C β-lactamases and D-alanyl-D-alanine-transpeptidases (DD-peptidases) [20,21].

**Figure 5:**
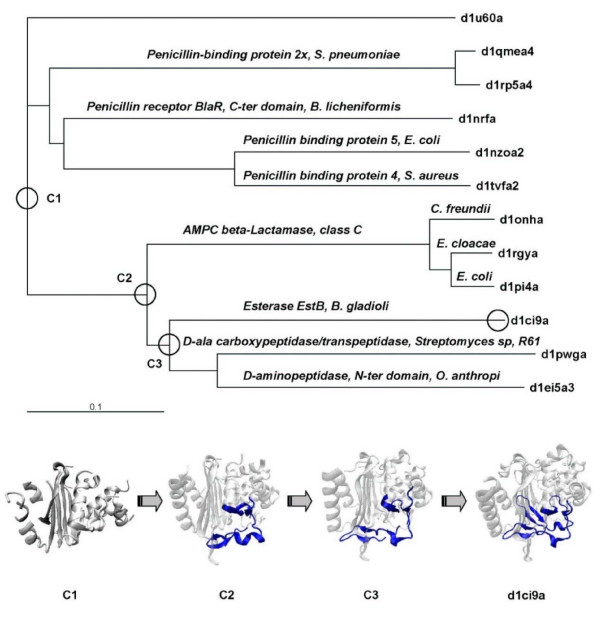
**The structure-based phylogenetic tree and the inferred evolutionary path of d1ci9a_**. C1, C2, and C3 are the inferred ancestral consensus structures on the evolutionary path of d1ci9a_. The variable region with nested inserts is shown in blue.

A novel nested insert was discovered on the EstB domain with insert rank of 3. The multiple sequence alignment of the variable region of all domains is given in Figure 4(a). The multiple structural alignment in Figure 4(b) shows the structural variation of different domains in the region. The structure-based phylogenetic tree of the family was built, and the insertions of d1ci9a_ were detected by comparing the structural consensus. The nested insertions of d1ci9a_ are color mapped according to the insert rank as shown in Figure 4(c). In Figure 5, the structure-based phylogenetic tree of the family was rooted with a domain d1u60a from the Glutaminase family, which also belongs to the β-lactamase/transpeptidase-like superfamily. The EstB domain d1ci9a_ are grouped with class C β-lactamases and DD-peptidases, and it is structurally closer to the DD-peptidases d1pwga. C1, C2, and C3 represent the common structures of clades marked with circles. C1 represents the ancestor and thus the core structure of the β-lactamase family. The inserts are shown in blue: By comparing the structures of C1 and C2, the first large insert can be identified as a segment with a small β hairpin and a short helix. In Figure 4(c), the insert is indicated in green on the d1ci9a_ variable region. From C2 to C3, the loop between the two β strands of the first insert region grows longer. This is the second nested insertion (d1ci9a_:233–237), the yellow segment in Figure 4(c). The third nested insert is an extra β-hairpin (d1ci9a_:242–261) of the EstB domain, which is red in Figure 4(c). It was inserted into the same variable region subsequently to the first hairpin insert in C3.

### Phylogenetic consistency of signal

**Figure 6:**
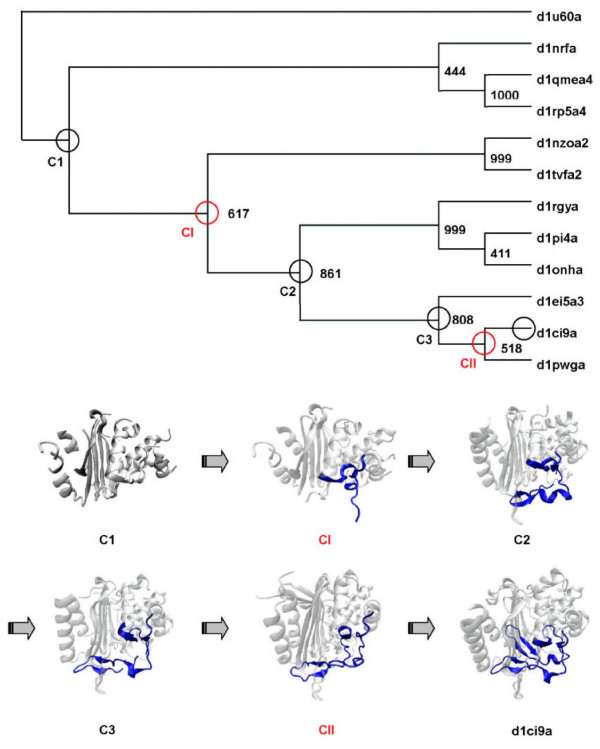
**The sequence-based phylogenetic tree and the inferred evolutionary path of d1ci9a_**. C1, CI, C2, C3, and CII are the inferred ancestral consensus structures on the evolutionary path of d1ci9a_. The variable region with nested inserts is shown in blue.

To validate the phylogeny and nested insertion events inferred with structure-based method, we also performed sequence-based phylogenetic analysis using the structure-based multiple sequence alignment (see Figure 6 and additional file 1). The unrooted sequence-based and structure-based trees are similar, and the stepwise growth of the same variable region is also detected in the sequence-based phylogeny. The topologies of the consensus trees inferred from bootstrap with distance method and the maximum likelihood method PHYML are consistent. A sequence-based phylogenetic tree is shown in Figure 6, and the confidence values of branches produced with bootstrap method in 1000 replicates is also shown. The main difference between the structure-based and sequence-based trees is the location of root. Given the inherent low sequence similarity within this dataset, a consistent "rooting" of this tree is not expected. The consensus structures of the node list from root C1 to d1ci9a suggest the iterative growth of the variable region along the path within the structure-based phylogeny (Figure 5). The consensus structures C1, C2, and C3 and their sequence in the node list of sequence-based phylogeny are the same as those of structure-based method. The sequence of ancestral consensus structures indicates a probable sequence of insertion events on the evolutionary path from root to the domain d1ci9a.

The EstB has a structural homology to β-lactamase, but shows no β-lactamase activity even though the nature and arrangement of active-site residues is very similar between EstB and the homologous β-lactamase. Modeling studies suggested steric factors account for the enzyme's selectivity for ester hydrolysis versus β-lactam cleavage [21]. One of the steric factors comes from the nested insert. The insert hairpin covers part of the active site entrance in EstB, which may affect the enzyme's selectivity by narrowing the access path to the active site tunnel. The stepwise nested insertions observed in the evolution of EstB demonstrate that the stepwise nested insertions can create novel complex sub-structure and affect the function of protein.

## Discussion

### Applicability of the structure-based phylogenetic method

For sequence-based phylogeny, manual editing of alignments is necessary to remove variable/gapped regions, and most methods cannot provide a reliable tree when the similarity amongst sequences is low. In contrast with sequence phylogenies, the substructures of a protein which are not suitable for sequence-based phylogeny actually constitute a source of phylogenetic signal. The structure-based phylogeny is thus expected to be more robust than sequence-based methods when domains in a family have low to very-low sequence similarities and regions which cannot be unambiguously aligned.

The SCOP database contains families with low sequence identity (<30%) despite having very close structures and functions [18]. These families are appropriate for tertiary structure phylogeny because more distant homologs usually have more insertions and structural variations. Several works have utilized structure-based methods to study these SCOP families with low sequence homology, including the β-Lactamase/D-ala carboxypeptidase family [19], the Class II aminoacyl-tRNA synthetase (aaRS)-like family [22], and the short-chain alcohol dehydrogenases family [23].

The structure-based distance metrics used in this work provide a reasonable estimate of phylogenetic distance. Several protein structural distance measures, including Root Mean Square Distances (RMSD) [19], and Hausdorff distance of loops [11,24], and *Q*_*H *_[12,13], have been applied to phylogenetic analysis. The distance metric *Q*_*H *_adopted in this work, which considers the differences of the aligned segments and the non-aligned gap regions simultaneously, has been successfully applied to study the evolution of structures in aminoacyl-tRNA synthetases [12], and has been built into the molecular modeling software VMD since version 1.8.3. Based on previous work and our results, the topologies of trees inferred with *Q*_*H *_distance are generally consistent with results of sequence-based methods. However, it is important to note that consistency with sequence-based phylogeny in difficult cases does not constitute a definitive proof of the suitability of *Q*_*H *_at capturing phylogenetic distances.

### Effect of flexible structural alignment

Many programs for the alignment of protein structures have been developed. Most structural alignment methods, such as CE [25] and DALI [26], assume that protein molecules are rigid bodies. This assumption is made despite the knowledge that many proteins are flexible molecules. The validity of the rigid body assumption is further questionable for variable regions since it is usually more flexible than the conserved core of the structure. When flexible molecules in different conformations are compared to each other as rigid bodies, even strong structural similarities can be missed. Several flexible structural alignment algorithms including FlexProt [27] and FATCAT [17] have been developed to solve the problem. In this study, flexible structural alignment method FATCAT was used to generate the pairwise structural alignment.

**Figure 7:**
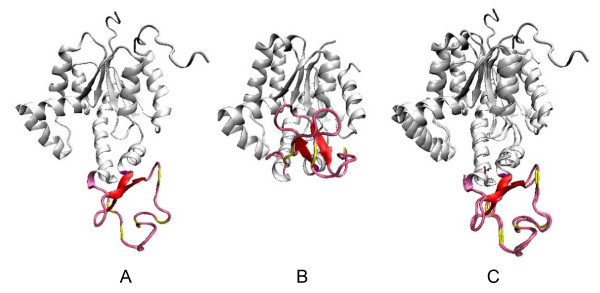
**Flexible structural comparison of protein 2ak3 and 1aky**. A. Protein 2ak3. B. Protein 1aky. C. Flexible structural alignment of 2ak3 and 1aky created by FATCAT. The flexible regions are aligned together.

Because the information used for phylogeny relies on structural distances, a reliable alignment of regions with higher structural variability is very important. Although some sub-structures may be homologs, the presence of a flexible hinge may cause a rigid body structural alignment method to fail to detect similarity. The structural alignment of protein 2ak3 and 1aky is such a case. The flexible regions of 2ak3 (125–161) and 1aky (131–168) have similar conformations but interact differently with the rest of the protein. The structures of the two domains and the flexible structural alignment created by FATCAT are illustrated in Figure 7. Because this type of signal is critical to our analyses, we used the flexible structural alignment algorithm FATCAT to generate the pairwise alignments of domain structures. The contributions of flexible structural alignment have been extensively discussed before [17,27,28].

### Accuracy of structure-based multiple sequence alignment

The accuracy of the structure-based multiple sequence alignments produced with the alignment tool developed for this study has been tested with a hard benchmark SABmark1.65 [29]. The average developer scores of our method for the superfamily and twilight sets in SABmark are 82.6 and 64.3, respectively. The average developer scores of T-Coffee are 57.7 and 27.4, respectively. The accuracy of the method proposed in this paper is thus sufficient to perform subsequent phylogenetic analyses on more difficult, sequence-based problems. The effect of using more accurate alignment methods on the phylogeny of distantly related sequence will be tested in the future.

There are a few databases providing protein structure-based alignments for homologous families, including HOMSTRAD [30] and PALI [31]. The structure alignment methods used by HOMSTRAD and PALI were COMPARER [32,33] and STAMP [34], respectively. In general, the structure-based sequence alignment can improve the accuracy of sequence alignment especially when sequences are distantly related. Both COMPARER and STAMP are rigid-body superposition programs. In this study, the multiple structure-based alignments of homologous families were produced by flexible alignment algorithm FATCAT. As discussed in the "Effect of flexible structural alignment" section, the flexible alignment method is very important for improving the accuracy of alignments.

## Conclusion

The signal provided by the tertiary structure of protein domains was used to infer phylogenies. It is important to contrast the nature of sequence and structural signals. The signal from structures is generated by the similarity of the shared regions (core) and the presence and magnitude of differences of their variable regions (loops). It is unclear whether the signal provided by the distance between the structures of the conserved cores reflects evolutionary distances. As cores are assumed to remain constant over time, the difference between sequences may be restricted to the presence and structure of the variable regions. An important caveat, our reconstruction of sequences of insertion did not account for the influence of deletion. Deletions in this context would appear as simultaneous insertions events in all other lineages. This would systematically increase the distance to all other related structures that are conserving the deleted segment. The effect of deletions on phylogenies would, therefore, bias this lineage toward the root of the tree.

It is clear that the underlying process of evolution of sequence and structure are different: homologous positions in sequences are subjected to a substitution process while their geometry is assumed to remain constant. The relative stability of structure over time, however, suggests that some of the tertiary structures contain phylogenetic signals that persist beyond the saturation of the substitution process of their coding sequence. The consequence to this is that 3D signal can be used to tackle questions spanning at unreachable evolutionary depths up to now.

One of these questions is whether a parsimonious process of innovation can be inferred from existing domain structures. As structural innovation is expected to be infrequent and spanning evolutionary distances which are not tractable at the sequence level, we constructed tertiary structure-based phylogenies. We reported that inserts which appear to have evolved iteratively are longer, more complex and some appear to be novel. For this reason, we propose that the methodology presented in this work is an early step to use tertiary structure phylogenetic analysis to study the evolution of structures and functional diversification.

## Methods

### Construction of test set

The test set of protein domain families was built from ASTRAL SCOP 1.69 domain subset (<95% sequence homology) [35]. The domains were sampled from the first six SCOP classes, including all alpha proteins, all beta proteins, alpha and beta proteins (a/b), alpha and beta proteins (a+b), multi-domain proteins, and the class of membrane and cell surface proteins and peptides. Other classes with either smaller size peptides or not a true class were excluded. The protein structure files in the domain list were then downloaded from the Protein Data Bank [36]. Domain structures were produced by extracting the polypeptide chains according to the domain definition of SCOP. When a SCOP domain was found discontinuous on one chain by definition, the segments in the middle of domain regions were kept.

Furthermore, domains with missing residues were excluded. In the final domain structure file, the amino acid sequence in the SEQRES records linearly corresponds to the ATOM records. To generate a rooted phylogenetic tree for the quantitative study of insertion history, a final filtering was performed on the domain set by selecting superfamilies that contained at least two families and at least one family with three or more domains. The immunoglobulin superfamily, which has 782 domains, was discarded because it is difficult to generate multiple sequence alignment for such large number of domains. After the filtering, the test set includes 3716 domains, belonging to 222 superfamilies, and 975 families in which there are 447 families with at least 3 domains.

### Phylogenetic tree

Flexible structural alignment was performed on every pair of domains in the superfamily using FATCAT [17]. From these pairs of aligned structures, a pairwise distance matrix was derived for each family using the structural distance measure *Q*_*H *_[12,13]. The structural distance measure *Q*_*H *_considers the structural distances of both the aligned core structures and the unaligned variable regions [12,13]. The structure-based tree was then generated using the Neighbor Joining algorithm [14]. To root the phylogenetic tree of each family, an outgroup domain was chosen from an adjacent structural family within the same superfamily.

### Structure-based multiple alignment

To produce an accurate multiple sequence alignment of a domain family for pinpointing insertion events, the structure-based multiple sequence alignment of each family was constructed with a similar method proposed by Casbon and Saqi in building S4, a database of structure-based sequence alignments of SCOP superfamilies [15]. This method generates high quality multiple structure-based alignment by running T-Coffee to perform hierarchical alignment using information from the pairwise structural alignments. T-Coffee has been successfully applied to incorporate sequence and structural information in building structure-based multiple sequence alignment [15,37,38]. In order to align the flexible regions of domains together, we used FATCAT [17] instead of SAP [39]. Our method includes the following procedures: 1. Run FATCAT to generate pairwise structural alignment for each pair of domains in a family; 2. Generate a T-Coffee library file from the pairwise structural alignments using the formula introduced by Casbon and Saqi [15]. A T-Coffee library file consists of the weights of equivalent residues in each pairwise structural alignment of all pairs of domains; 3. Run T-Coffee (version 2.66) [16] to produce a multiple sequence alignment from the library file.

### Detect insertions and nested insertions

**Figure 8:**
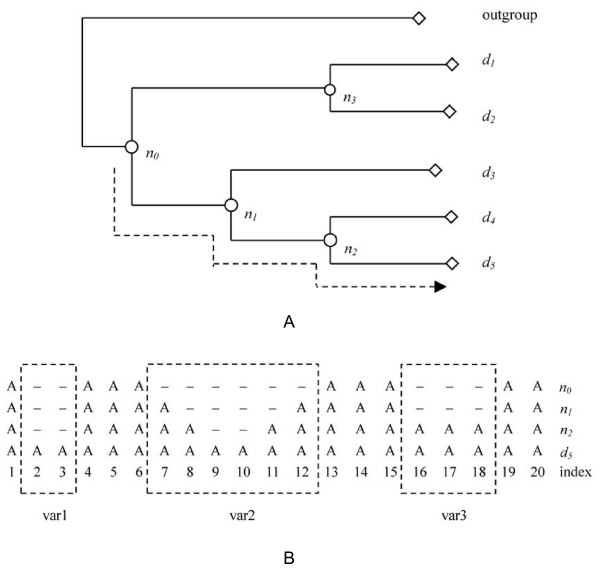
**Schematic diagram for detecting insertion and nested insertion using the ancestral structural consensus**. A. A phylogenetic tree rooted with an outgroup domain. *d*_1 _~ *d*_5 _represent the homologous domains in a domain family, and *n*_0 _~ *n*_3 _represent the ancestral structural consensus. B. Detect insertion and nested insertion using the aligned character sequences of the ancestral structural consensus, which are grouped together according to evolutionary stages from *n*_0 _to *d*_5_. In the aligned sequences, a site with 'A' denotes over 90% structures of all descendant domains have aligned residues at this site, otherwise the site is denoted as a gap '-'. var1, var2, and var3 are three insertion-containing regions determined with the aligned sequences, in which var2 is a variable region with nested inserts.

Because the phylogenies are rooted, it is possible to identify at which node in the tree a site enters the conserved core for all descending structures. Given a phylogenetic tree, with a set of internal nodes *N *= {*n*_1_,..., *n*_*M*-2_}, relating to a set of *M *domains *D *= {*d*_1_,...*,d*_*M*_}, an evolutionary path is defined as a sequence of nodes starting from the root *n*^0 ^to the *i*th leaf (domain), denoted as *p*_*i *_= {*n*^0^,..., *n*^*k*^,..., *d*_*i*_}, in which *n*^(*k*) ^∈ *N*. The ancestral structural consensus for internal node *n*^*k *^is inferred by comparing the structures of all descendant domains. A site of the consensus is marked as "A" if over 90% of the structures in the structure-based multiple sequence alignment of all the descendant domains have aligned residues at this site; otherwise the site is denoted as a gap ('-') (Figure 8). *n*^0 ^is the consensus sequence of all descendent domains. A variable region is defined as a segment of consecutive gap sites with size >1 on *n*^0 ^bounded by the shared consensus sites, which are denoted as "A". In Figure 8(b), the character sequences of the ancestral structural consensus of the nodes in the path *p*_5 _are grouped together to infer the sequence of insertions in domain *d*_5_. By comparing the structural consensus of the nodes, a segment is considered as an insertion at *n*^*k *^if it has no homolog in the sequence of *n*^*k*-1^. To minimize the effect of alignment artifact, small insertions with only one site were ignored. Therefore, only insertions with two or more residues were considered in this study. A new insertion at *n*^*k *^was considered as nested insertion if it was bounded by sites previously identified as inserts during the traversing from *n*^0 ^to *n*^*k*-1^. In Figure 8(b), three insertion-containing regions, var1, var2, and var3, can be detected. The insertion between *n*_2 _and *n*_1 _at site 8 and 11 in var2 is a nested insertion because it is bounded by the earlier insertion at site 7 and 12 between *n*_1 _and *n*_0_. In contrast, the insertions in var1 and var3 are non-nested, *e.g. *block insertions.

### Insert rank

Insert rank is a value to define the nesting depth of insertions. The insert rank of a nested insert is a value greater than 1. The insert rank of the nested insert var3 in Figure 8(b) is 3, which indicates three insertions are observed. For the non-nested inserts of var1 and var3, the insert rank is 1.

### Mapping of insertions into structures

Insertions were mapped into domain's original 3D coordinates for visualization. The temperature factors/beta values of insert residues in the PDB format file were modified with scaled values to be consistent with the insert rank. After mapping, the color of residues in the insert region shifts from blue (rank 1) to red (the highest rank).

### Determine complex and novel inserted sub-structure

To study the potential of an insert to be a novel fold unit, we define the structural complexity as follows: a sub-structure is complex if the similarity to itself is considered significant. Sub-structures whose self similarity is not significant are too simple to be considered (e.g. a small segment of helix, a very short polypeptide, etc.). We restricted the evaluation of complexity to block inserts with rank = 1, length ≥ 10 and nested inserts with rank >2, length ≥ 10. Every insert extracted from the SCOP domain was aligned against its source domain using FATCAT. An insert was then considered complex if the P-value of the structural alignment was <0.05. The P-value determined by FATCAT is a reasonable threshold to assign significance in structural similarity [28]. The assignments of secondary structures for the complex inserts were analyzed with Stride [40,41].

To investigate whether there was any direct evidence to show insertions created novel structures, all the complex inserts were compared to domains from other superfamilies in the ASTRAL SCOP 1.69 domain subset (<95% sequence homology) using FATCAT. The alignment of a significant match is defined as P-value < 0.05 and aligned length >80% of the insert size. An insert will be considered as a novel structural unit if there is no significant match of the nested insert in the SCOP domain subset.

### Other methods

The statistical distributions of inserts (Figure 1 and Figure 2) were generated using R [42]. The phylogenetic trees were rendered with TreeView 1.6.6 [43]. The illustrations of protein structures were prepared with VMD [44] and Pymol [45]. Sequence-based phylogenetic analysis was performed with both distance-based method provided by PHYLIP [46] and maximum likelihood method PHYML [47,48]. The sequence-based phylogenies were built with PROTDIST program and JTT substitution matrix. The programs SEQBOOT and CONSENSE were used to estimate the confidence of branches from 1000 bootstrap replicates. The maximum likelihood phylogeny was built with PHYML using the JTT substitution matrix and keeping all other options to the default setting using the PHYML web server [48]. The annotated multiple sequence alignment of Figure 4(a) was generated using JalView [49].

## Authors' contributions

HJ developed the methodology, analyzed results, and wrote the manuscript. CB conceived of the study and wrote the manuscript. All authors read and approved the final manuscript.

## Supplementary Material

Additional file 1 Figure S1. Sequence based phylogenetic tree of the β-Lactamase/D-ala carboxypeptidase family calculated with Phyml. Click here for file

Additional file 2 Figure S2. Another example of the nested insertions detected in the domain d1m56b1 within the Periplasmic domain of cytochrome c oxidase subunit II family (SCOP family 49541). A. The structure-based phylogenetic tree. B. The variable region of domain d1m56b1 with insert rank of 3. Inserts are color coded according the insert rank. C. The superimposed variable regions of the 6 domains. D. Sequence alignment of the variable region. Click here for file

Additional file 3 Figure S3. The multiple structural alignment of the six domains in the Periplasmic domain of cytochrome c oxidase subunit II family. Residues are color-coded from blue to red according the sequence conservation. Blue: most conserved; Red: most variable. The sequence conservation score of a residue is a scaled value of the number of residues aligned on the site in the structure-based multiple sequence alignment. Click here for file
