# Supplementary material for: Insertions and the emergence of novel protein structure: a structure-based phylogenetic study of insertions
Source: BMC Bioinformatics. 2007 Nov 15;8:444. doi: 10.1186/1471-2105-8-444 (PMC2225427; doi:10.1186/1471-2105-8-444)

Figure S1. Sequence based phylogenetic tree of the β-Lactamase/D-ala carboxypeptidase family generated using Phyml.

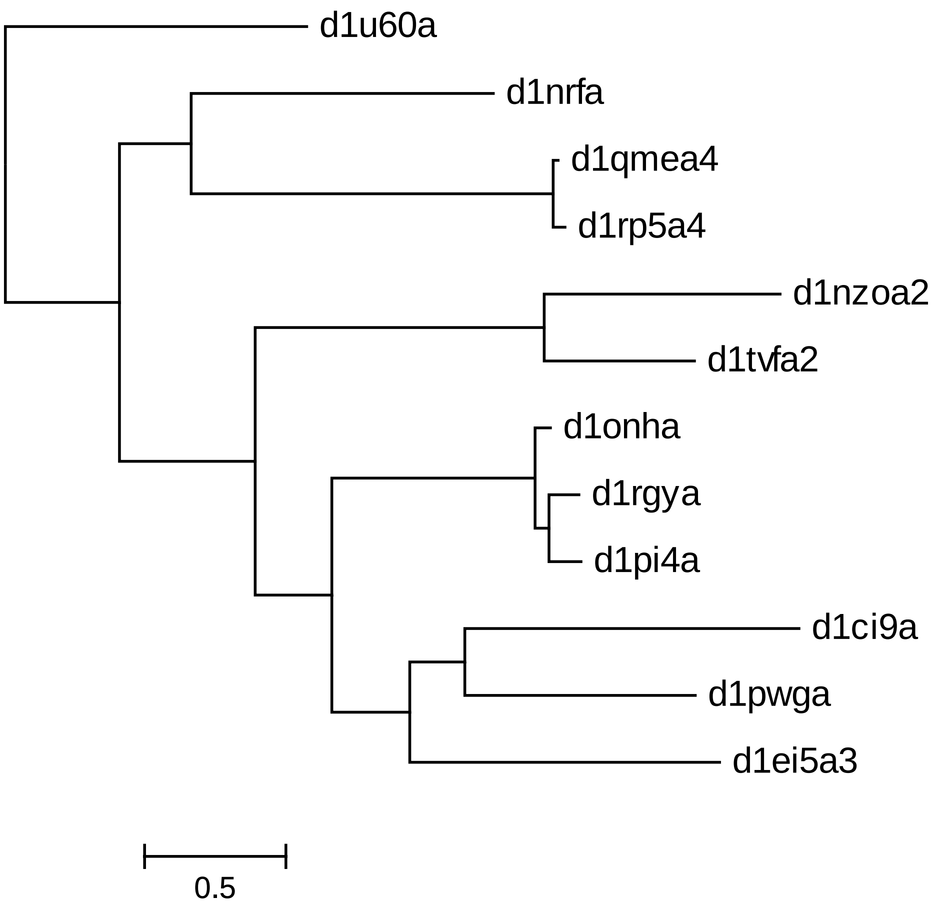

Supplement: Additional file 1 — Figure S1. Sequence based phylogenetic tree of the β-Lactamase/D-ala carboxypeptidase family calculated with Phyml. [file 1471-2105-8-444-S1.pdf]
