# Supplementary figures and images for: Insertions and the emergence of novel protein structure: a structure-based phylogenetic study of insertions
Source: BMC Bioinformatics. 2007 Nov 15;8:444. doi: 10.1186/1471-2105-8-444 (PMC2225427; doi:10.1186/1471-2105-8-444)

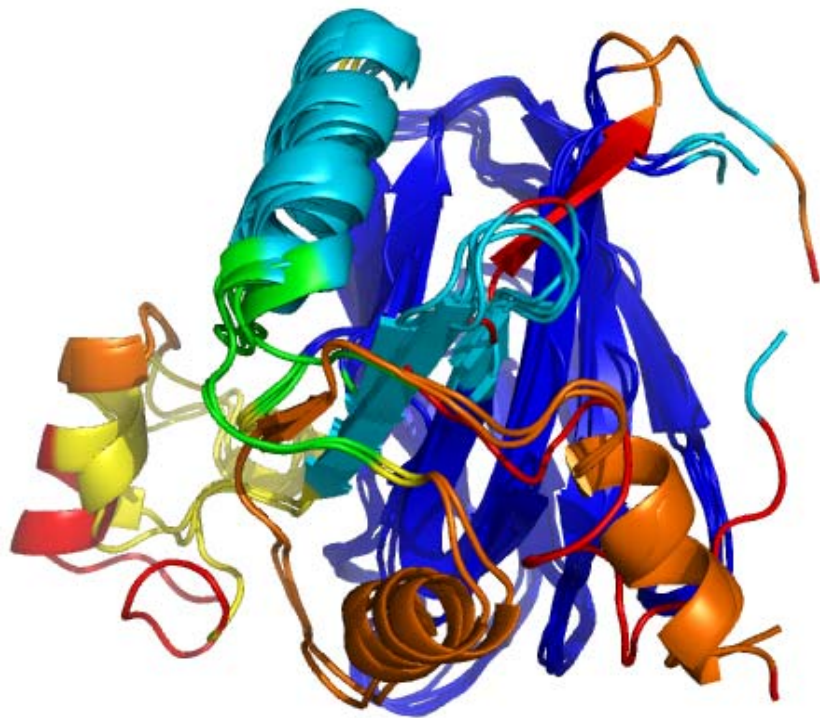

Supplement: Additional file 3 — Figure S3. The multiple structural alignment of the six domains in the Periplasmic domain of cytochrome c oxidase subunit II family. Residues are color-coded from blue to red according the sequence conservation. Blue: most conserved; Red: most variable. The sequence conservation score of a residue is a scaled value of the number of residues aligned on the site in the structure-based multiple sequence alignment. [file 1471-2105-8-444-S3.pdf]
