# Supplementary material for: Insertions and the emergence of novel protein structure: a structure-based phylogenetic study of insertions
Source: BMC Bioinformatics. 2007 Nov 15;8:444. doi: 10.1186/1471-2105-8-444 (PMC2225427; doi:10.1186/1471-2105-8-444)
Supplement: Additional file 2 — Figure S2. Another example of the nested insertions detected in the domain d1m56b1 within the Periplasmic domain of cytochrome c oxidase subunit II family (SCOP family 49541). A. The structure-based phylogenetic tree. B. The variable region of domain d1m56b1 with insert rank of 3. Inserts are color coded according the insert rank. C. The superimposed variable regions of the 6 domains. D. Sequence alignment of the variable region. [file 1471-2105-8-444-S2.pdf]

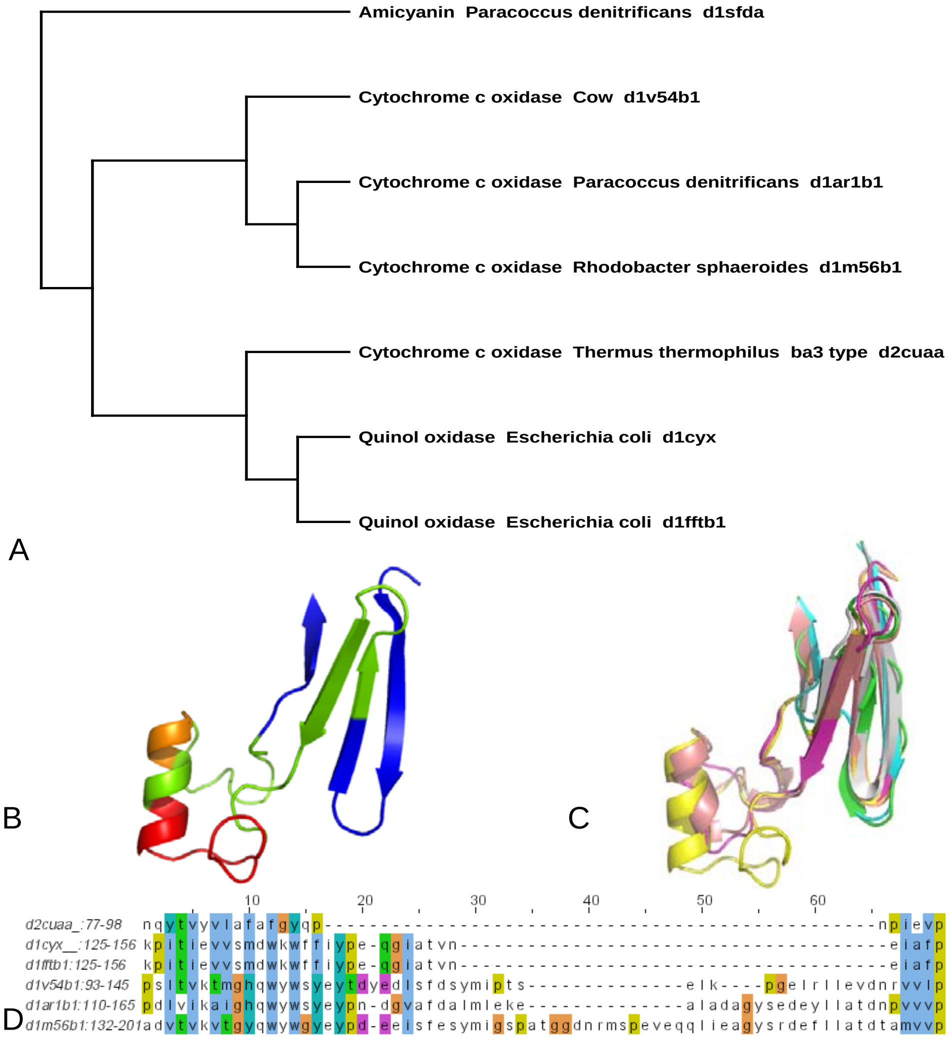

Amicyanin Paracoccus denitrificans d1sfda
Cytochrome c oxidase Cow d1v54b1
Cytochrome c oxidase Paracoccus denitrificans d1ar1b1
Cytochrome c oxidase Rhodobacter sphaeroides d1m56b1
Cytochrome c oxidase Thermus thermophilus ba3 type d2cuaa
Quinol oxidase Escherichia coli d1cyx
Quinol oxidase Escherichia coli d1fftb1
A
B
C
D
10 20 30 40 50 60
d2cuaa_:77-98 nqytvyvlafafgyqp---npievp
d1cyx__:125-156 kpitievvsmdwkwffiype-qgiatvn--------------------------------------eiafp
d1fftb1:125-156 kpitievvsmdwkwffiype-qgiatvn--------------------------------------eiafp
d1v54b1:93-145 psltvktmghqwywsyeytdyedlsfdsymipts--------------elk----pgelrllevdnrvvlp
d1ar1b1:110-165 pdlvikaighqwywsyeypn-dgvafdalmleke--------------aladagysedeyllatdnpvvvp
d1m56b1:132-201 advtvkvtgyqwywgyeypd-eeisfesymigspatggdnrmspeveqqlieagysrdefllatdtamvvp
